# Huoxin Pill Attenuates Cardiac Inflammation by Suppression of TLR4/NF-*κ*B in Acute Myocardial Ischemia Injury Rats

**DOI:** 10.1155/2020/7905902

**Published:** 2020-07-09

**Authors:** Jianfeng Chu, Xueling Zhou, Meizhong Peng, Yan Lu, Ali Farman, Lianli Peng, Huajian Gao, Qi Li, Xintong Chen, Lingling Xie, Youqin Chen, Aling Shen, Jun Peng

**Affiliations:** ^1^Academy of Integrative Medicine, Fuzhou, Fujian 350122, China; ^2^Chen Keji Academic Thought Inheritance Studio, Fuzhou, Fujian 350122, China; ^3^Fujian Key Laboratory of Integrative Medicine on Geriatrics, Fujian University of Traditional Chinese Medicine, Fuzhou, Fujian 350122, China; ^4^College of Engineering and Computer Science, The Australian National University, Canberra, ACT 2600, Australia; ^5^Department of Research and Development, Youcare Pharmaceutical Group Co., Ltd., Beijing 100176, China; ^6^Department of Pediatric Gastroenterology, Rainbow Babies & Children's Hospital, Case Western Reserve University School of Medicine, Cleveland, OH 44106, USA

## Abstract

Huoxin Pill (HXP), a traditional Chinese medicine, has been prescribed widely in the treatment of coronary heart disease, angina pectoris, and other diseases. However, the possible protective mechanisms of HXP on myocardial ischemia remain unclear. In the current study, we investigated the effects and potential mechanism of HXP on myocardial ischemia and cardiac inflammation and the activation of TLR4/NF-*κ*B pathway. Determination of electrocardiogram, echocardiography, and heart weight index (HWI) indicated that HXP treatment obviously attenuated the elevation of ST-segment, end-diastolic volume, and HWI in the AMI rat model. Enzyme-linked immunosorbent assay (ELISA) demonstrated that Huoxin Pill treatment significantly decreased the levels of CTnT, CK-MB, MDA, IL-6, and TNF-*α*, while it increased SOD content in serum of the AMI rat model. Moreover, hematoxylin and eosin (HE) and immunohistochemistry (IHC) staining revealed that HXP treatment alleviated pathological change, infiltration of inflammatory cells, levels of IL-6 and TNF-*α*, and expression of TLR4 and p-NF-*κ*B in cardiac tissues of the AMI rat model. In conclusion, HXP treatment significantly improves cardiac function and attenuates cardiac inflammation by suppressing the activation of TLR4/NF-*κ*B pathway in the ISO-induced AMI rat model. This study provides insights into the potential of HXP on prevention and treatment of AMI.

## 1. Introduction

Acute myocardial ischemia (AMI), a pathological myocardial state resulting from imbalance between myocardial oxygen demand and coronary blood supply [1, 2], is a common symptom of coronary heart disease and other cardiac diseases such as cardiogenic shock, free wall rupture, ventricular septal rupture, acute mitral regurgitation (MR), and other critical complications [3]. Increasing evidences indicate that AMI is one of the leading causes of morbidity and mortality worldwide by resulting in reduction of oxygen supply to the heart, myocardial energy metabolism dysfunction, and failure to support the normal work of the heart [4, 5]. As the symptoms continue to develop, the disability, recurrence, and mortality rate will increase and pose great threat to the life and health of patients [6, 7]. Despite the development of aggressive high-potency medicine therapies and global cardiovascular risk reduction efforts, the related significant cardiovascular risk still exists [8]. Therefore, it is urgent to develop novel strategies for heart protection during AMI.

Recent studies on myocardial infarction mechanisms suggest that oxidative stress and inflammatory response play essential roles in the pathogenesis of myocardial infarction [9, 10]. Increase of reactive oxygen species during AMI leads to the occurrence of oxidative stress injury, resulting in myocardial cell death and tissue damage [11, 12]. When inflammatory response occurs after myocardial infarction, inflammatory cytokines are released and the numbers of macrophages gradually increase, provoking further myocardial damage [13]. However, antioxidant stress response and anti-inflammatory may prevent these harmful events and attenuate myocardial dysfunctions [14, 15]. In the pathophysiological processes of myocardial ischemia, heart injury can be aggravated due to the large number of inflammatory factors and oxygen free radicals [16], leading to critical syndromes such as arrhythmia and heart failure, and initiating the activation of many signaling pathways [17]. Among these pathways, the Toll-like receptors (TLRs) serve as pattern-recognition receptors at a proximal step in the innate immune response to inflammatory response [18].

Continuous expression of TLR4, one of the major TLRs expressed by myocardial cells, was enhanced significantly in the myocardium after acute myocardial ischemia injury and participated in signaling pathways, particularly implicated in proinflammatory response by activating NF-*κ*B pathway [19, 20]. NF-*κ*B is identified as a crucial player during the development of AMI [21, 22]. Activation of NF-*κ*B leads to the transcription of inflammation cytokines, including TNF-*α* and IL-6, which further promotes inflammation [23]. These studies suggest that targeting TLR4/NF-*κ*B pathway-modulated inflammation can be a novel strategy for AMI treatment.

Due to the complex pathogenic mechanisms of AMI, it is a challenge to treat AMI using a single-molecule approach. In contrast, traditional Chinese medicine treatment (TCM) with its unique compatibility advantages has been standardized and advocated in clinical practices [24]. It has shown unique advantages and significant curative effects on improving clinical symptoms of coronary heart disease and patient life quality through multitargets and multifunctions [25, 26]. As a traditional Chinese formula, Huoxin Pill (HXP) is composed of ginseng, *Ganoderma lucidum*, aconite, bezoar, bear bile, toad venom, borneol, musk, safflower, and pearl, which is mainly used for coronary heart disease, angina pectoris, and other diseases. Many components in HXP, such as ginsenoside, muscone, and bufodienolide, have been shown to possess antimyocardial ischemia activities [27–29]. However, the possible protective mechanisms of HXP on myocardial ischemia remain unclear. Therefore, the aim of the current study was to investigate the cardioprotective effects of HXP against acute ischemic myocardial injury in an ISO-induced rat model and explore the effect of HXP on TLR4/NF-*κ*B signaling pathway.

## 2. Materials and Methods

### 2.1. Drugs and Reagents

Isoproterenol was purchased from Shanghai Hefeng Pharmaceutical Co., Ltd. (Shanghai, China). ELISA kits for rat IL-6 (Cat No. MM-0190R1), rat TNF-*α* (Cat No. MM-0180R1), rat CK-MB (Cat No. MM-0625R1M), rat MDA (Cat No. MM-0385R1), and rat SOD (Cat No. MM-0386R1) were all purchased from Jiangsu Enzyme Free Industrial Co., Ltd. (Jiangsu, China). Anti-IL-6 antibody (Cat No. 21865-1-AP) was obtained from Proteintech Group, Inc. (Wuhan, Hubei, China). Anti-NF-*κ*B P65 (phospho-Ser536) antibody (Cat No. YP0191) was purchased from ImmunoWay Biotechnology Company (Plano, TX, USA). Antibody for anti-TLR4 (Cat no. SAB-35463) was purchased from SAB (College Park, Maryland, USA). Antibodies for Anti-NF-*κ*B P65 (Cat No. GTX107678) and Anti-TNF-*α* (Cat No. GTX35134) were obtained from GeneTex (San Antonio, Texas, USA).

### 2.2. Preparation of HXP

HXP (concentrated pill; Z44021835) was provided by Youcare Pharmaceutical Group Co., Ltd. (Guangzhou, Guangdong, China). HXP was grinded into a powder and dissolved in a corresponding amount of distilled water to a final concentration of 3 mg/kg/day or 9 mg/kg/day (1.5 ml for each rat) based on the body weight of rats just before use.

### 2.3. Experimental Animals

All animal experiments were performed strictly in accordance with “Guide for the Care and Use of Laboratory Animals” and were approved by the Institutional Animal Care and Use Committee of Fujian University of Traditional Chinese Medicine (Approval No. 2020011). The male Wistar rats (age: 6 weeks; *n* = 32) with body weight 180–220 g were obtained from Beijing Vital River Laboratory Animal Technology Co., Ltd. (Beijing, China). All rats were housed in specific pathogen-free (SPF) rooms with controlled temperature (22°C), humidity (50–60%), and a 12 h light/dark cycle. Food and water were provided ad libitum throughout the experiment.

### 2.4. Experimental Design and Treatment

The rats were randomly divided into 4 groups (*n* = 8 each group): Sham, Acute myocardial ischemia (AMI), HXP-L, and HXP-H groups. Rats in HXP-L and HXP-H groups were administered with 3 mg/kg/d and 9 mg/kg/d of HXP for 10 days, respectively; rats in Sham and AMI groups had received an equivalent volume of distilled water. At the 9th and 10th day, the rats in AMI, HXP-L, and HXP-H groups were injected subcutaneously with isoproterenol (8 mg/kg/day), while the rats in the Sham group were injected with an equivalent volume of saline [30].

### 2.5. Determination of ST-Segment Elevation and Cardiac Function

The electrocardiogram (ECG) test was conducted in anesthetized rats before the first injection and after the final injection of ISO or saline. The needle electrodes were linked to the four-limb skin and chest of the rats, and the electrocardiographic patterns were recorded with an ECG (VE-300, EDAN, Shenzhen, China) recording and analysis system within 10 min after final injection of ISO or saline [16].

### 2.6. Cardiac Echocardiography

Following the ECG test, transthoracic echocardiography was performed using a Vevo 2100 Ultrasound machine (VisualSonics, Toronto, Ontario, Canada). Briefly, rats were anesthetized with isoflurane, maintaining heart rate at 300 to 350 beats per minute. Then, the rats were placed in dorsal recumbency on a heated platform. The images were acquired in the two-dimensional mode under the parasternal long-axis view. Analysis of image was performed using the Vevo Strain Software (Vevo LAB 1.7.1). At the end of the experiment, the hearts were dissected and cardiac tissues were stored in liquid nitrogen or fixed with 4% paraformaldehyde for further use.

### 2.7. ELISA Analysis

The serum levels of CTnT, CK-MB, SOD, MDA, IL-6, and TNF-*α* of rats in each group were determined using ELISA analysis. Briefly, an equal volume (10 *μ*l) of serum from each group was used to determine the levels of these proteins according to the manufacturer's instructions. At the end of reaction, optical density (OD) value was measured at 450 nm wavelength using Microplate Reader (Tecan, Männedorf, Switzerland). The concentration was calculated based on the standard curve.

### 2.8. Hematoxylin and Eosin (HE) and Immunohistochemical Staining

Cardiac tissues were fixed with 4% paraformaldehyde for 24 h and then soaked in 75% ethanol at room temperature [31]. Cardiac tissues were embedded in paraffin and sliced into 5 *μ*m thick section, after that dewaxed and dehydrated section were stained with hematoxylin and eosin (H&E) to detect the histopathology of cardiac tissues. In addition, immunodetection was performed to detect the expression of TNF alpha (1 : 400), IL-6 (1 : 200), TLR4, NF-*κ*B-P65 (1 : 400), and NF-*κ*B- P65 (phospho-Ser536) (1 : 200). Briefly, slides were incubated with the above primary antibodies overnight at 4°C. Then, the slices were washed and incubated with HRP-polymer conjugated anti-rabbit IgG second antibody (Maixin, Fujian, China), followed by the reaction using DAB kit (Maixin) and counterstaining of Hematoxylin. All of the sections were examined by a light microscope (LEICA, Wetzlar, Germany) at a magnification 400x; six views of each section were randomly selected to analyze the expression of these protein. The positive rates were semiquantitatively analyzed using the Motic 6.0 image analysis system (Motic, Xiamen, China).

### 2.9. Statistical Analysis

All data are expressed as mean ± standard deviation (SD) and all statistical analyses were performed using SPSS 22.0 (SPSS Inc.). One-way analysis of variance was performed to compare statistical significance among ≥ 3 groups when the data met normal distribution. *p* < 0.05 indicates statistical significance.

## 3. Results

### 3.1. HXP Treatment Attenuates the Elevation of ST-Segment in AMI Rats

**Figure 1:**
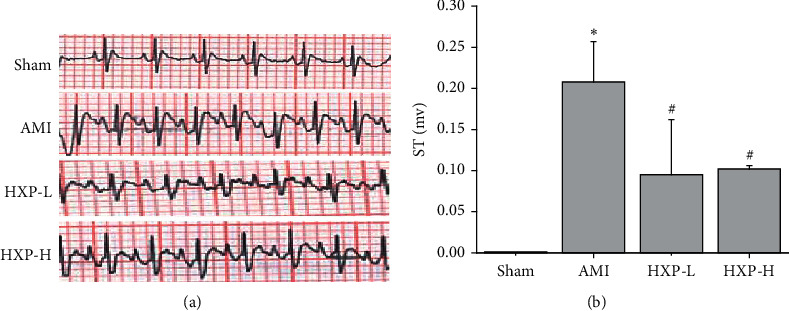
ECG was performed to detect the ST-segment elevation. (a) The representative images of ECG from each group. (b) ST-segment elevation was analyzed in each of group (Sham, AMI, HXP-L, and HXP-H groups). Data are represented by mean ± SD. ^*∗*^*p* < 0.05 vs. Sham group. ^#^*p* < 0.05 vs. AMI group.

ISO was injected subcutaneously and significantly elevated ST-segment in AMI rats was observed as compared to the Sham group, which demonstrate the successful establishment of acute myocardial ischemia injury model (Figures 1(a) and 1(b)). With the treatment of HXP, the ST-segment elevation was significantly attenuated in the HXP-L and HXP-H groups while compared with the AMI group (Figures 1(a) and 1(b)).

### 3.2. HXP Treatment Alleviates the Increase of End-Diastolic Volume in AMI Rats

**Figure 2:**
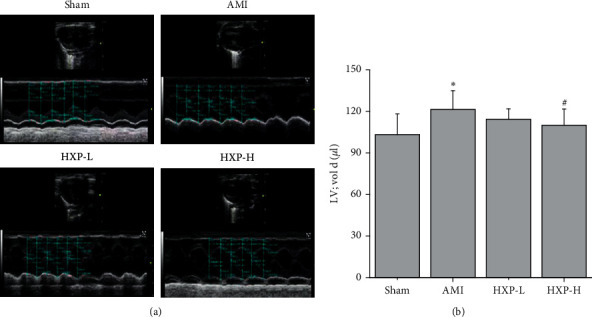
Echocardiography was performed to detect the LV diastolic function. (a) The representative images of echocardiography from each group. (b) The LV diastolic function of rats was analyzed. Data are represented by mean ± SD. ^*∗*^*p* < 0.05 vs. Sham group. ^#^*p* < 0.05 vs. AMI group.

Echocardiography was performed to determine the cardiac diastolic and systolic functions of rats in each group. As shown in Figures 2(a) and 2(b), compared with Sham group (LV; Vol; d:103.24 ± 14.97 *μ*L), the end-diastolic volume of rats in AMI group (LV; Vol; d:121.50 ± 13.45 *μ*L) was significantly increased, which obviously attenuated after 9 mg/kg of HXP treatment (LV; Vol; d: 110.04 ± 11.66 *μ*L).

### 3.3. HXP Treatment Reduces Myocardial Injury Markers CTnT and CK-MB Levels in AMI Rats

**Figure 3:**
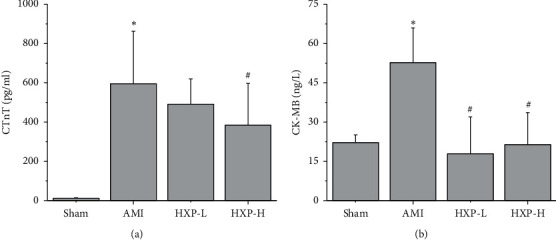
ELISA was performed to detect the level of cardiac marker. ELISA was performed to detect the level of cardiac marker enzymes CTnT (a) and CK-MB (b) in serum. Data are represented by mean ± SD. ^*∗*^*p* < 0.05 vs. Sham group. ^#^*p* < 0.05 vs. AMI group.

ELISA analysis was performed to determine the alterations of myocardial injury markers (CTnT and CK-MB) in the serum of rats in each group. As two major indicators of myocardial injury marker enzymes, CTnT and CK-MB levels were significantly increased in serum of AMI rats (^*∗*^*p* < 0.05, vs. Sham group), which were dramatically reduced after HXP treatment (^#^*p* < 0.05, vs. AMI group) (Figures 3(a) and 3(b)).

### 3.4. HXP Treatment Reduces MDA Levels and Elevates SOD Levels in AMI Rats

**Figure 4:**
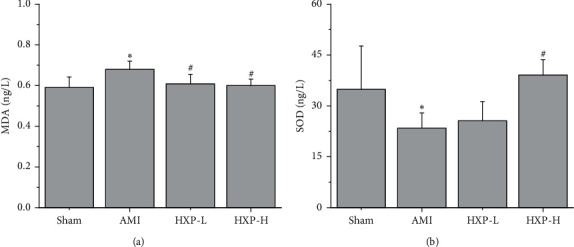
ELISA was performed to detect the levels of MDA and SOD in serum. ELISA was performed to detect the levels of both MDA (a) and SOD (b) in serum. Data are represented by mean ± SD. ^*∗*^*p* < 0.05 vs. Sham group. ^#^*p* < 0.05 vs. AMI group.

The levels of MDA and SOD were measured in the serum of rats in each group by ELISA analysis. As shown in Figure 4(a), ISO injection obviously increased the levels of MDA in serum of AMI rats, which was alleviated after HXP treatment (^*∗*^*p* < 0.05, vs. Sham group; ^#^*p* < 0.05, vs. AMI group). In contrast, ISO injection obviously decreased the levels of SOD (Figure 4(b); ^*∗*^*p* < 0.05, vs. Sham group), which were increased after 9 mg/kg of HXP treatment (Figure 4(b); ^#^*p* < 0.05, vs. AMI group).

### 3.5. HXP Treatment Reduces Serum Levels of Proinflammatory Cytokines IL-6 and TNF-*α* in AMI Rats

**Figure 5:**
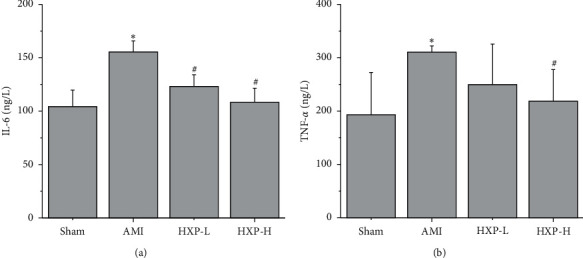
ELISA was performed to detect the levels of inflammatory cytokines. ELISA was performed to detect the level of IL-6 (a) and TNF-*α* (b) in serum. Data are represented as mean ± SD. ^*∗*^*p* < 0.05 vs. Sham group. ^#^*p* < 0.05 vs. AMI group.

The serum levels of proinflammatory cytokines IL-6 and TNF-*α* were detected by ELISA analysis. As shown in Figure 5(a), the serum levels of IL-6 were obviously increased after ISO injection (^*∗*^*p* < 0.05, vs. Sham group), which were obviously reduced after HXP treatment (^#^*p* < 0.05, vs. AMI group). Similarly, HXP treatment significantly reduced the elevation of TNF-*α* levels in serum of AMI rats (Figure 5(b), ^*∗*^*p* < 0.05, vs. Sham group; ^#^*p* < 0.05, vs. AMI group).

### 3.6. HXP Treatment Attenuates Elevation of Heart Weight and Pathological Changes in AMI Rats

**Figure 6:**
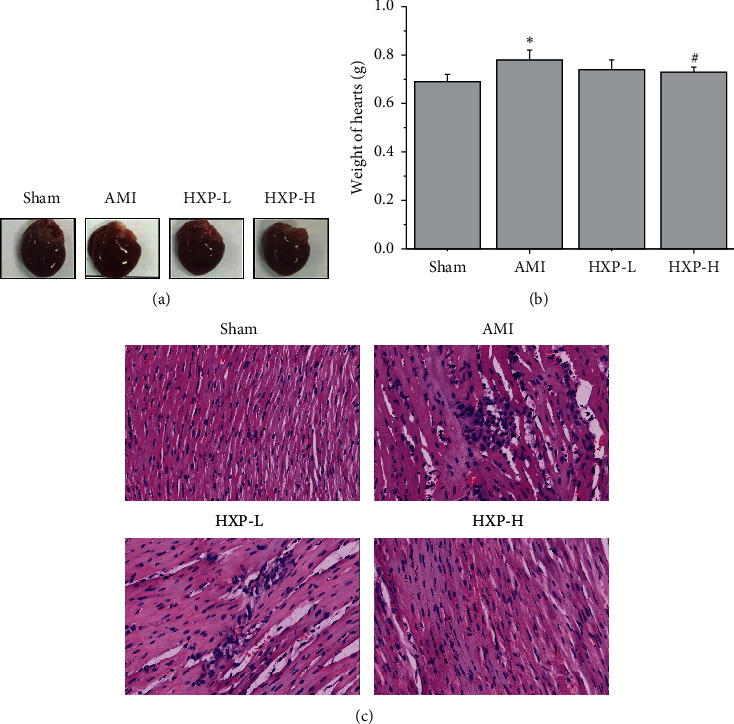
Heart weight and HE staining were performed to assess the heart pathological changes. (a) The representative image of heart from each group. (b) The heart weight was measured in each group (Sham, AMI, HXP-L, and HXP-H groups). Data are represented by mean ± SD. ^*∗*^*p* < 0.05 vs. Sham group. ^#^*p* < 0.05 vs. AMI group. (c) Morphologic changes of cardiac tissue were detected by HE staining in all groups of rats (Sham, AMI, HXP-L, and HXP-H groups) at a magnification of ×400.

Determination of heart weight revealed that ISO injection significantly increased the weight of heart (Figures 6(a) and 6(b); ^*∗*^*p* < 0.05, vs. Sham group), compared with the Sham group. However, after administration of HXP (9 mg/kg), the heart weight was significantly decreased (Figures 6(a) and 6(b); ^#^*p* < 0.05, vs. AMI group). HE staining was further performed to observe the pathological changes of cardiac tissues in each group. As shown in Figure 6(c), we observed a normal myofibrillar structure, branched appearance, and continuity of cells in cardiac tissues of Sham rats, while cardiac tissues of rats from the AMI group exhibited apparent myocardial cell edema, degeneration, transverse striations loss, and increased inflammatory cell infiltration, which were significantly attenuated after administration of HXP.

### 3.7. HXP Treatment Reduces IL-6 and TNF-*α* Cytokine Expression in Cardiac Tissues of AMI Rats

**Figure 7:**
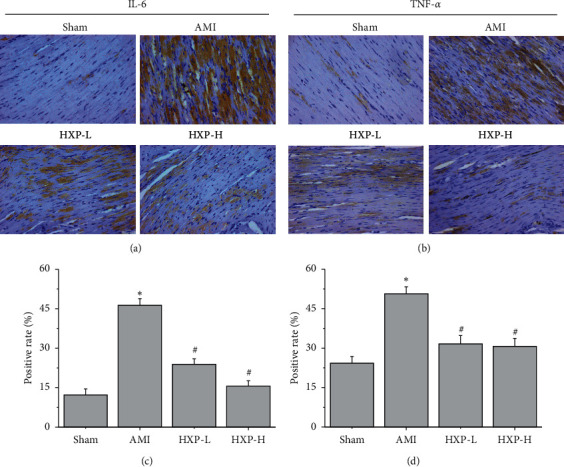
IHC analysis was performed to determine the protein expression of inflammatory cytokines. IHC analysis was performed to determine the protein expression of IL-6 and TNF-*α* expression in cardiac tissue. The representative images of IL-6 (a) and TNF-*α* (b) from each group were acquired at a magnification of ×400. The quantification of IL-6 (c) and TNF-*α* (d) protein levels was analyzed. Data are represented as mean ± SD. ^*∗*^*p* < 0.05 vs. Sham group. ^#^*p* < 0.05 vs. AMI group.

Immunohistochemistry analysis was used to determine the expression of IL-6 and TNF-*α* in cardiac tissues of rats in each group. As shown in Figures 7(a)–7(d), immunohistochemistry analysis revealed that the cytokine expressions of both IL-6 and TNF-*α* in cardiac tissues were significantly increased in AMI group (^*∗*^*p* < 0.05, vs. Sham group). On the contrary, HXP treatment obviously decreased the cytokine expression of IL-6 and TNF-*α* in cardiac tissues (^#^*p* < 0.05, vs. AMI group).

### 3.8. HXP Treatment Downregulated TLR4 and p-NF-*κ*B Expression in Cardiac Tissues of AMI Rats

**Figure 8:**
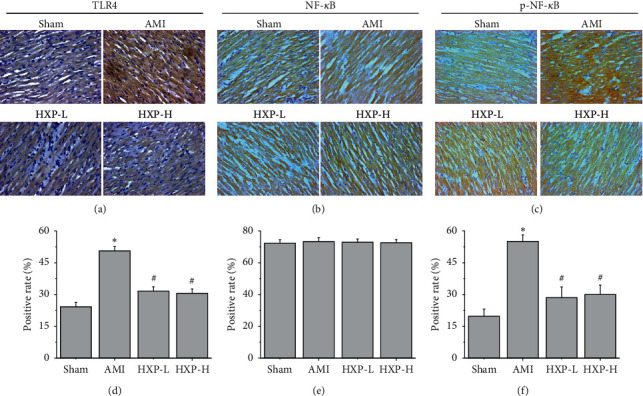
IHC analysis was performed to determine TLR4 and p-NF-*κ*B expression. IHC analysis was performed to determine the protein expression of TLR4, NF-*κ*B, and p-NF-*κ*B. The representative images of TLR4 (a), NF-*κ*B (b), and p-NF-*κ*B (c) were acquired at a magnification of ×400. The quantification of TLR4 (d), NF-*κ*B (e), and p-NF-*κ*B (f) was analyzed. Data are presented as mean ± SD. ^*∗*^*p* < 0.05 vs. Sham group. ^#^*p* < 0.05 vs. AMI group.

To further study the mechanism of HXP on attenuation of ISO-induced AMI, immunohistochemistry analysis was performed to detect the expression of TLR4, NF-*κ*B, and p-NF-*κ*B in cardiac tissues. As shown in Figures 8(a)–8(f), the protein expression of TLR4 was significantly upregulated in cardiac tissues of AMI rats (^*∗*^*p* < 0.05, vs. Sham group), while it was obviously downregulated after HXP treatment (^#^*p* < 0.05, vs. AMI group). Further determination of NF-*κ*B expression did not find difference among different groups. However, we found obvious upregulation of phospho-NF-*κ*B (p-NF-*κ*B) expression in cardiac tissues of AMI rats (^*∗*^*p* < 0.05, vs. Sham group), which was attenuated after HXP treatment (^#^*p* < 0.05, vs. AMI group).

## 4. Discussion

The complex pathogenic mechanisms of AMI and limitation of current treatment highlight the importance of TCM in AMI treatment. Due to its unique compatibility advantages, TCM had been widely used in clinical practices in AMI treatment [24], facilitating unique curative effects on improving clinical symptoms of coronary heart disease and patient life quality [25, 26]. Because HXP is mainly used for coronary heart disease, we explored the role of HXP in the treatment of AMI for the first time. Using ISO-induced AMI rat model, we found that HXP treatment significantly attenuated the elevation of ST-segment, end-diastolic volume, and heart weight of AMI rats. Consistent with the above studies, further determination of myocardial injury markers CTnT and CK-MB levels by ELISA analysis revealed that HXP treatment reduced the increment of both CTnT and CK-MB levels in serum of ISO-induced AMI rats. Moreover, upon determination of pathological changes, apparent myocardial cell edema, degeneration, and transverse striations loss were observed, which were attenuated after HXP treatment. These studies indicated that HXP treatment obviously attenuated ISO-induced cardiac dysfunction in AMI rats. However, the protective effects of HXP on cardiac function should be further confirmed in other AMI animal models.

Due to the essential role of oxidative stress and inflammatory responses in the pathogenesis of myocardial infarction [9, 12], antioxidant stress and anti-inflammatory responses present novel strategies on harmful event prevention and myocardial dysfunction attenuation [14, 15]. Therefore, the current study determined the effects of HXP treatment on serum levels of antioxidant enzyme SOD, the lipid peroxidation marker MDA, and proinflammatory cytokines (IL-6 and TNF-*α*). ELISA analysis demonstrated that HXP treatment significantly alleviated the elevation of MDA, IL-6, and TNF-*α* levels and the reduction of SOD levels in serum of ISO-induced AMI rats. Moreover, HE and IHC staining showed apparent increase of inflammatory cell infiltration and protein levels of both IL-6 and TNF-*α* in cardiac tissues of AMI rats, which were significantly alleviated after HXP treatment. Collectively, HXP treatment reduced ISO-induced oxidative stress and inflammatory responses in AMI rats, which might be one of the mechanisms of HXP on cardiac protection. However, the pathophysiological process of AMI involves several mechanisms: inflammation, reactive oxygen species production, apoptosis, and intracellular calcium overload [32–34]. Therefore, the effects of HXP on these processes need to be further addressed in future studies.

TLR4/NF-*κ*B is a well-known signaling pathway and plays a critical role in the meditation of inflammatory responses and the pathogenesis of AMI via regulation of proinflammatory cytokine synthesis [19–23], including TNF-*α* and IL-6, which further promotes inflammation [23]. More importantly, HXP reduced the increase of TNF-*α* and IL-6 in AMI rats, which encourage us to further explore the regulatory effect of HXP on activation of TLR4/NF-*κ*B pathway in cardiac tissues of AMI rats. As expected, IHC analysis revealed that the protein expression of TLR4 and p-NF-*κ*B was significantly upregulated in cardiac tissues of AMI rats and obviously was downregulated after HXP treatment without affecting the expression of NF-*κ*B. These studies suggested that HXP exerts its cardioprotective effects by inhibiting the inflammatory response through regulating the TLR4/NF-*κ*B signaling pathway. However, the effect of HXP on translocation of p-NF-*κ*B and other downstream effectors should be further addressed.

## 5. Conclusions

The current study suggests the potential critical cardiac protective role of Chinese traditional medicine HXP in alleviating oxidative stress injury and inflammatory reaction of myocardium caused by acute myocardial ischemia. Furthermore, the important mechanism of signaling pathway is regulated by TLR4/NF-*κ*B in the myocardium. However, more details are needed to explore the various clinical approaches of HXP and determine the effect of this medicine at molecular level to treat different pathological conditions.

## Data Availability

All data generated or analyzed during this study are included within the article and available from the corresponding author upon request.
